# HMOX1 is partly responsible for phenotypic and functional abnormalities in mesenchymal stem cells/stromal cells from placenta of preeclampsia (PE) patients

**DOI:** 10.1186/s13287-020-1557-6

**Published:** 2020-01-21

**Authors:** Yasser S. Basmaeil, Dana Algudiri, Reem Alenzi, Abdullah Al Subayyil, Ayodele Alaiya, Tanvir Khatlani

**Affiliations:** 1 Stem Cells and Regenerative Medicine Department, King Abdullah International Medical Research Center, King Abdulaziz Medical City, Ministry of National Guard Health Affairs, P.O. Box 22490, Mail Code 1515, Riyadh, 11426 Saudi Arabia; 2 Stem Cell and Tissue Re-Engineering Program, King Faisal Specialist Hospital and Research Centre, PO Box: 3354, Riyadh, 11211 Saudi Arabia

**Keywords:** Preeclampsia, Decidua basalis mesenchymal stem cells, xCELLigence, Preconditioning, Tin protoporphyrin, HMOX1

## Abstract

**Background:**

Preeclampsia is a common obstetric syndrome affecting women in their first pregnancy and characterized by hypertension and proteinuria, which appears after 20 weeks of gestation. It is characterized by high blood pressure and occasional damage to another organ system most often the liver and kidneys. Currently, the etiology and pathogenesis of this syndrome are not fully understood. Since mesenchymal stem cells/stromal cells (MSCs) are intimately associated with endothelial cells that line vessel walls in the decidua they may play some role in the pathogenesis of this syndrome. In this study, we have partly, unveiled the mechanism of preeclampsia pathogenesis at the stem cells level.

**Methods:**

We have isolated and characterized MSCs from decidua basalis of preeclampsia placenta (PE-DBMSCs) and showed their decreased functionality in terms of proliferation, migration, adhesion and clone formation potential as compared to MSCs isolated from decidua region of normal placentae (DBMSCs). The cells were preconditioned with H_2_O_2_ and the functional characteristics were evaluated. Differentially expressed genes were analyzed using mass spectrometry. Immunoblotting confirmed the expression of these proteins.

**Results:**

Pre-conditioning with H_2_O_2_ restored the functional outcome of PE-DBMSCs. Mass spectrometry (MS) analysis of differentially expressed proteins revealed HMOX1 as one of the major candidates missing in PE-DBMSCs. HMOX1 inhibition by tin protoporphyrin (SnPP) in normal DBMSCs resulted in a reduction in proliferation, migration, adhesion, and clone formation processes as compared to the untreated controls. mRNA and protein analyses of PE-DBMSCs preconditioned with H_2_O_2_ at lower doses showed upregulation of HMOX1 expression.

**Conclusions:**

We hereby show for the first time that loss of function of stem cells/stromal cells isolated from the patients with preeclampsia may contribute towards the disease exacerbation. Our results suggest that HMOX1 may be partially responsible for the loss of functionality in PE-DBMSCs and contribute significantly towards the pathophysiology of preeclampsia. However, further investigation is required to decipher its exact role in the development and onset of the disorder.

## Introduction

Preeclampsia (PE) affects about 3–7% of pregnant women worldwide. Among various causes of pregnancy-related deaths, especially in the developing world, PE remains the number one cause of death in pregnant women [1]. It is usually observed during the second trimester of pregnancy. PE is associated with various clinical symptoms, which include hypertension and proteinuria, which are more prominent after 20 weeks of gestation [2]. Although the actual cause of this disorder is still unclear, factors arising from the placenta are usually blamed. The only corrective measure available so far is early fetal termination and removal of the placenta. Strong evidence points towards maternal endothelial dysfunction, which is supposed to be responsible for its early signs [3]. Causative factors of endothelial dysfunction include poor placental vascular remodeling, oxidative stress [4], excessive inflammation [5], and variation in expression of angiogenic factors [6]. Angiogenic factors seem to be more robustly involved in clinical signs and severity of the disorder [7–10]. An increase in inflammation is not necessarily observed before the onset of the disease, yet an increase in inflammation is the hallmark of severe PE but is not the real cause of the disorder [11, 12]. Since mesenchymal stem cells/stromal cells (MSCs) are intimately associated with endothelial cells that line vessel walls in the decidua, they might play some role in the pathogenesis of this disorder.

MSCs (stromal cells) isolated from the maternal side of the human term placenta, also called *decidua basalis* (DBMSCs), have exclusive characteristic features. They have shown to prevent inflammation in various inflammatory diseases [13]. Exposure to hydrogen peroxide (H_2_O_2_) enhanced survival, proliferation, adhesion, and migration of DBMSCs [14]. Furthermore, preconditioning with H_2_O_2_ upregulated expression of genes responsible for improving cellular functionalities and downregulated expression of specific genes with opposing effects on their functional outcome [14].

Oxidative stress caused by stimuli, such as modified lipids, hypoxia, hyperoxia, and ischemia, upregulate the expression of heme oxygenase (HMOX) [15]. HMOX is expressed in two isoforms, HMOX1 and HMOX2. HMOX1 degrades heme into biliverdin, free iron, and carbon monoxide (CO) [16]. Biliverdin is reduced to bilirubin with anti-oxidant properties, whereas CO has anti-apoptotic properties [17]. HMOX is involved in several biological processes that regulate oxidative stress, apoptosis, and inflammation [18]. HMOX1 protects cardiac stem cells from apoptosis. It is involved in the proliferation of breast [19] and pancreatic cell lines [20]. Besides, HMOX1 is found overexpressed in prostate cancer, brain tumors, and melanomas [21–24].

Here, we report the isolation and characterization of MSCs (stromal cells) from *decidua basalis* of the placenta from human PE patients (PE-DBMSCs) using our previously published methods [13]. Our aim is to understand if placental mesenchymal stem cells/stromal cells could be involved in the onset of the disorder, and the underlying mechanism behind their dysfunction. PE-DBMSCs showed decreased functionality concerning proliferation, migration, adhesion, and clone formation potential as compared to MSCs isolated from the decidua region of normal placentae (DBMSCs). Pre-conditioning with H_2_O_2_ restored the functional outcome of PE-DBMSCs. Mass spectrometry analyses identified HMOX1 as one of the major candidates missing in PE-DBMSCs. It has been reported that deficiency of HMOX1 resulted in endothelial damage [25], recurrent miscarriages [26], retardation of intrauterine growth [27], and PE [28].

Inhibition of HMOX1 protein resulted in a reduction in proliferation, migration, adhesion, and clone formation processes in DBMSCs as compared to the controls, proving that HMOX1 may be partially responsible for the loss of functionality in PE-DBMSCs. The involvement of HMOX1 in stem cells/stromal cells isolated from PE patients has not been investigated yet. Therefore, the aim of this study is to elaborate on the mechanism of the loss of functionality of the PE-DBMSCs, and here we provide a possible evidence demonstrating the role of HMOX1 and stem cells/stromal cells at the onset of PE.

## Material and methods

### Ethical approval and collection of human placentae

The Institutional Review Board (IRB) at King Abdullah International Medical Research Centre, Riyadh, Saudi Arabia, approved this study. Human placentae from patients with confirmed cases of PE (diagnosed with a moderate and severe level of disease status as per the international standards) and with uncomplicated pregnancies through normal vaginal delivery (38–40 weeks of gestation) were collected after informed consent from the patients. The gestational age and fetal viability of normal pregnancies were confirmed by early ultrasound examination before 20 weeks of gestation. All placentae were processed within 2 h of delivery.

### Isolation and culture of mesenchymal stem cells/stromal cells

DBMSCs from normal placentae and PE-DBMSCs from the placenta obtained from PE patients were isolated from the *decidua basalis* region that remains attached to the human term placenta after delivery, as previously described [13]. Briefly, 10 g of the tissue was dissected from the maternal surface of the placenta and washed thoroughly with sterile phosphate-buffered saline (PBS, pH 7.4) to remove excess blood. The tissue was then finely minced and washed thoroughly with PBS. After centrifugation at 300×*g* for 5 min, the tissue pellet was digested using collagenase digestion solution containing 0.3% collagenase type I (Life Technologies, Grand Island, USA) diluted in PBS, 100 μg/mL streptomycin and 100 U/mL penicillin, 271 unit/mL DNase I (Life Technologies) at 37 °C in a water bath for 1 h. The mixture was then filtered with a 100-μm nylon filter (Becton Dickinson, NJ, USA), centrifuged and incubated with red blood cell lysing buffer (#sc-3621, FCM Lysing solution, Santa Cruz, CA, USA) at room temperature (RT) for 45 min to lyse red blood cells. After centrifugation, cells were washed and cultured in 75cm^2^ flask (Becton Dickinson) in complete DBMSC culture medium containing Dulbecco’s modified Eagle’s medium nutrient mixture F-12 9 (DMEM-F12), 10% mesenchymal stem cell certified fetal bovine serum (MSC FBS) (Life Technologies), 100 μg/mL L-glutamate, 100 μg/mL streptomycin and 100 U/mL penicillin. Cells were then incubated at 37 °C in a humidified atmosphere containing 5% CO_2_ and 95% air (a cell culture incubator). When cells reached 75% confluence, they were harvested with TrypLE express detachment solution (Life Technologies), and characterized by flow cytometry using MSC-positive markers (CD44, CD90, CD146, CD166, and CD105) and hematopoietic-negative markers (CD19, CD40, CD45, CD80, CD86, and HLA-DR) as previously described [13].

### Mesenchymal stem cell/stromal cell differentiation

Before any further experimental uses, the MSCs at passage 3 were assessed for differentiation into adipocytes, chondrocytes, and osteocytes as previously described [13]. Adipogenic, osteogenic, and chondrogenic differentiation were performed by incubating DBMSCs in adipogenic (#390415), osteogenic (#390416), and chondrogenic (#390417) media, respectively. All differentiation media were purchased from R&D Systems (Abingdon, UK). Each differentiation medium was supplemented with 10% MSCFBS, 100 μg/ml of L-glutamate, 100 μg/ml streptomycin, and 100 U/l penicillin. Adipocytes, osteoblasts, and chondrocytes were identified using LipidTOX™ Green, Alizarin Red S, and Alcian Blue dyes, respectively, as previously described [13]. All antibodies were from Beckman Coulter (CA, USA). Thirty placentae were used in this study. Cells from passage 3–5 were used in subsequent experiments.

### Antibodies and reagents

GAPDH and a panel of fluorescent-labeled antibodies used for flow cytometry were purchased from R&D Systems (Minneapolis, MN, USA). HMOX1 antibody was purchased from AbCam (Cambridge, MA, USA) and ANXA6, HSP71, and β-Actin antibodies were purchased from Cell Signaling Technology (Danvers, MA, USA). Goat anti-rabbit and goat anti-mouse secondary antibodies were purchased from Sigma-Aldrich (St. Louis, MO, USA). SnPP (cat. no. 0747) was purchased from Tocris Bioscience (Minneapolis, MN, USA). Protease inhibitor cocktail was from Millipore Sigma (Burlington, MA, USA).

### Flow cytometry

PE-DBMSCs were phenotypically characterized by flow cytometry as described previously [13]. Cells were harvested as described above and 1 × 10^5^ of cells were stained with monoclonal antibodies (CD19, CD40, CD44, CD45, CD80, CD86, CD90, CD146, CD166, and CD105, HLA-DR, IL-1β, IL-12, TNF-α, ICAM-1, CXCL4, and CXCR4) for 30 min and then washed twice with cold PBS by centrifugation at 150×*g* for 5 min at 8 °C. To analyze the intracellular expression of molecules, cells were fixed with 4% paraformaldehyde in sterile PBS, pH 7.4 for 10 min at RT and then permeabilized using sterile PBS, pH 7.4 containing 0.1% saponin for 5 min at RT. The expression of corresponding intracellular and cell-surface proteins were assayed by BD FACS CANTO II (Becton Dickinson) flow cytometer. As negative controls, cells in a separate tube were treated with FITC or PE-labeled mouse IgG or IgM antibody.

### H_2_O_2_ treatment

PE-DBMSCs and DBMSCs were cultured in complete DBMSC culture medium containing hydrogen peroxide (H_2_O_2_), (30% (w/w), Sigma Aldrich, USA) at a final concentration of 50 μM and 100 μM and incubated at 37 °C in a cell culture incubator. Cells were incubated for 72 h (pre-treatment conditions) and/or treated simultaneously with 50 μM and 100 μM (in-treatment conditions) of H_2_O_2_ while performing the functional assays, as per the previously standardized protocols [14].

### Cell proliferation assay using a tetrazolium compound [3-(4, 5-dimethylthiazol-2-yl)-5-(3-carboxymethoxyphenyl)-2-(4-sulfophenyl)-2H-tetrazolium, inner salt]

To examine the proliferation of PE-DBMSCs the cells were seeded at a density of 5 × 10^3^ per well in 96-well tissue culture plates containing complete DBMSC culture medium and incubated for 24 h at 37 °C in a cell culture incubator. Cell proliferation was evaluated using a MTS kit (#G5421, CellTiter 96® Aqueous Non-Radioactive Cell Proliferation Assay, Promega, Germany) according to the manufacturer’s instructions. Briefly, MTS solution was added into each well of the 96 well assay plate containing DBMSCs in complete culture medium with or without H_2_O_2_, and incubated at 37 °C in a cell culture incubator. After 4 h, the absorbance at 490 nm was recorded using an ELISA plate reader (Spectra MR, Dynex Technologies, Denkendorf, Germany), and results were presented as means (±SD) obtained from triplicate samples. MTS solution in a medium not exposed to cells was also used as blank. Experiments were performed with triplicate samples and repeated five times using cells from passages 3–5 of five independent preparations of PE-DBMSCs.

### Migration assay using RTCA

PE-DBMSC and DBMSC cellular migration was examined using the xCELLigence Real-Time Cell Analyzer (RTCA-DP version; Roche Diagnostics, Mannheim, Germany), which continuously monitors cellular events by recording label-free changes in electrical impedance (reported as cell index) as previously described [29]. We used 16-well plates (#05665825001, CIM-16, Roche Diagnostics) as previously described, with minor modifications [29]. The CIM plates have 16-well migration chambers comprising upper and lower chambers separated by a porous (pore size 8 μm) polyethylene terephthalate (PET) membrane in conjunction with microelectrodes. Treatments with SnPP or with H_2_O_2_ were made to desired concentrations (to a final volume of 160 μL) and loaded in the lower wells of the plate. Following the addition of 50 μL pre-warmed media to the wells of the upper chamber, the plates were locked in the RTCA DP device at 37 °C in a cell culture incubator for 1 h to obtain equilibrium as per the manufacturer’s instructions and a measurement step was then performed as a background signal, generated by cell-free media. To initiate the experiment, the untreated or treated (pre- and in-treated with 50 μM and 100 μM H_2_O_2_) PE-DBMSCs and DBMSCs were seeded at a density of 20 × 10^3^ in the upper chamber in 100 μL and the plates were incubated at RT for 30 min to allow the cells to settle onto the membrane as per the manufacturer’s instructions. DBMSC medium supplemented with 20% FBS was added to the lower chamber. Each test was performed with quadruplicate samples and after equilibration; the analyzer was programmed to scan the membrane every 15 min for 24 h. The impedance value of each well was automatically monitored by the xCELLigence system for 24 h and expressed as a CI value. Experiments were performed with quadruplicate samples and repeated as described above. Migration observed in the presence of 20% FBS, and with medium alone, served as positive and negative controls, respectively.

### Proliferation and adhesion assay using RTCA

The xCELLigence system (RTCA-DP version; Roche Diagnostics, Mannheim, Germany) was used to determine the adhesion and proliferation of PE-DMBSCs and DBMSCs after preconditioning, and in DBMSCs after HMOX1 inhibition. Briefly, 100 μL complete medium was added to each well in 16-well culture plate, E-Plate 16 (catalog number 05469813001, Roche Diagnostics), and the background impedance was achieved as previously described [29]. Treated and untreated control cells were seeded in quadruplicate wells, and equilibrium was achieved by leaving the culture plates for 30 min at RT before data recording. The culture plates were placed in the xCELLigence system at 37 °C in a cell culture incubator and cell index was monitored for 72 h. Data was analyzed by the RTCA xCELLigence software (version 1.2.1). For cell adhesion, data was measured after 2 h, whereas the rate of cell proliferation was calculated after 24, 48, and 72 h and after normalization with the adhesion data. Data is expressed as cell index with mean and standard errors. All experiments were performed in triplicate.

### Colony-forming unit assay

Colony-forming efficiency of untreated or treated (pre- and in-treated with 50 and 100 μM H_2_O_2_) was evaluated as previously described [14]. Briefly, untreated or treated cells were harvested, washed with PBS, seeded into six-well plates at a density of 100 cells/well in a complete DBMSC culture medium, and incubated at 37 °C in a cell culture incubator. Untreated cells were used as control. The cells were fed with fresh medium every 3 days. After 14 days of culture, the medium was removed and the cells washed with PBS, and fixed with 4% paraformaldehyde in PBS, pH 7.4 at RT for 30 min. After washing with PBS, the cells were stained with 0.1% Crystal Violet (Sigma-Aldrich. MO, USA) at RT for 15 min, rinsed with distilled water, visualized, and photomicrographs were recorded as described above. Colonies of cell aggregates of ≥ 50 cells were scored. Experiments were performed with triplicate samples and repeated as described above.

### Real-time PCR

Total RNA was isolated from (untreated or treated cells) using the RNEasy mini kit (Qiagen, MD, USA). Four micrograms of RNA was transcribed into the single-stranded cDNA using the Fastlane cDNA Analysis Kit (Qiagen, MD, USA). Real-time PCR reaction was performed in triplicate on the CFX96 real-time PCR detection system (Bio-Rad, CA USA). Data were analyzed using the CFX manager software (Bio-Rad, CA USA). The results were exported to Microsoft Excel for further analysis. The results are expressed in terms of fold change by calculating the ΔΔ^−2^ values. The expression of internal housekeeping gene was used as a loading control. Each experiment was performed on PE-DBMSCs (passage 3) prepared from three individual placentae.

### Mass spectrometry analyses

Prior to protein identification, approximately 40 μg total protein extracts were loaded on to 1-D SDS-PAGE for micro preparative mass spectrometry analysis. The interested protein bands on the 1-D gel were excised from colloidal Coomassie blue-stained gels. Five gel plugs from each band of interest were excised by robotic Spot Cutter (Bio-Rad, CA USA) and deposited into a 96-well microtiter plate. In-gel protein digestion was carried out 37 °C for 4.5 h by Janus™ Automated Mass Prep Station (Perkin Elmer, USA) as previously described [30]. The digested peptides were diluted to achieve a concentration of approximately 1 μg/μL prior to loading on to the column for LC/MS^E^ analysis.

Protein identification was performed using one-dimensional Nano Acquity Liquid Chromatography coupled with tandem mass spectrometry on Synapt G2 HDMS (Waters, Manchester, UK). All sample analyses were done on a Triazaic Nano source (Waters, Manchester, UK) in the positive ion mobility mode nanoESI as previously described with minor modifications [30, 31]. Briefly, a total of 3 μL sample injection representing approximately 3 μg protein digests for each sample was loaded on HSS T3 column and samples were infused using the Acquity Sample Manager with mobile phase comprising of A1 (99% water + 1% acetonitrile + 0.1% Formic acid) and B1 100% Acetonitrile + 0.1% formic acid with high sample flow rate of 0.450 μL/min.

Data-independent acquisition (MS^E^)/ion mobility separation experiments were performed, and data was acquired over a range of m/z 30–200 Da with a scan time of 1 s, ramped transfer collision energy 20–50 V with a total acquisition time of 120 min. All samples were analyzed in triplicate runs (triplicate runs were repeated on two different occasions as a measure of reproducibility) and data were acquired using the Mass Lynx programs (version. 4.1, SCN833, Waters, Manchester, UK) operated in resolution and positive polarity modes. The acquired MS data were background-subtracted, smoothed, and de-isotoped at medium threshold.

The Protein Lynx Global Server (PLGS) version 2.5 was used for all automated data processing and database searching using the Uniprot species-specific database (uniprot.org) for protein identification (Waters, UK). The data was filtered to show only unambiguous protein identification using multiple parameters including expected molecular mass, percentage coverage, number of peptides and PLGS scores.

### Immunoblotting

Untreated or treated cells were washed twice with cold sterile PBS, pH 7.4 to remove debris. Following the addition of 100 μL of cell lysis buffer (Cell Signaling Technologies, Beverly, MA) containing protease and phosphatase inhibitors to each well, cells were scraped using a cell scrapper and lysate was collected, centrifuged at 15,000 rpm for 5 min at 4 °C to remove the debris, and the supernatant was then collected. Total protein was estimated by Bradford assay method. Briefly, after mixing 30 μg of extracted proteins with an equal amount of 2X Laemmli sample buffer (Bio-Rad, Hercules, CA, USA), boiled for 7 min and then loaded onto a 10% sodium dodecyl sulfate-polyacrylamide gel (SDS-PAGE, Bio-Rad, Hercules, CA, USA). The separated proteins were transferred onto a nitrocellulose membrane using Mini Trans blot system (Bio-Rad, Hercules, CA, USA) at 120 V for 100 min. After blocking the membranes for 30 min at RT in Tris-buffered saline with 0.1% (v/v) Tween 20 (TBS-T) containing 5% non-fat dry milk (Bio-Rad, Hercules, CA, USA), the membranes were incubated with primary antibodies at 1:1000 dilutions overnight at 4 °C. The next day, the membranes were washed three times with TBS-T and incubated with horseradish peroxidase (HRP)-conjugated secondary antibodies (R&D Systems, Minneapolis MN) at 1:3000 dilution for 2 h at RT. After washing three times with TBS-T, specific protein bands were visualized using SuperSignal™ West Pico or West Femto Chemiluminescent Substrate (Thermo Fisher Scientific, Waltham, MA) in a ChemiDoc visualization system (Bio-Rad, Hercules, CA, USA). Densitometry of the bands was performed by the image analyzing software Image Lab (Bio-Rad, Hercules, CA, USA). The results were normalized with the values obtained for β-Actin. Each experiment was performed in triplicate using cells from passages three to five, from five individual placentae.

### Statistical analysis

Data are presented as means ± standard deviation (SD) of at least three independent experiments each performed with at least triplicate samples. Student’s *t* test was used for comparisons and a *p* value of < 0.05 was considered to be statistically significant.

## Results

### Isolation and characterization of PE-DBMSCs

PE-DBMSCs were isolated from the *decidua basalis* attached to the maternal side of the human placenta and these cells formed a homogenous monolayer of adherent cells, which exhibited a fibroblast-like cell morphology. Contamination by fetal derived cells in the PE-DBMSC populations was assessed by RT-PCR detecting for the SRY gene. The RT-PCR of all PE-DBMSCs isolated from 20 placentae did not detect the SRY gene, confirming that these cell preparations consisted only of maternal-derived cells. The positive control was the detection of the SRY gene in the placental mesenchymal stem cell preparation of fetal-derived cells as previously published [13] (data not shown).

### Differentiation of PE-DBMSCs

**Fig. 1:**
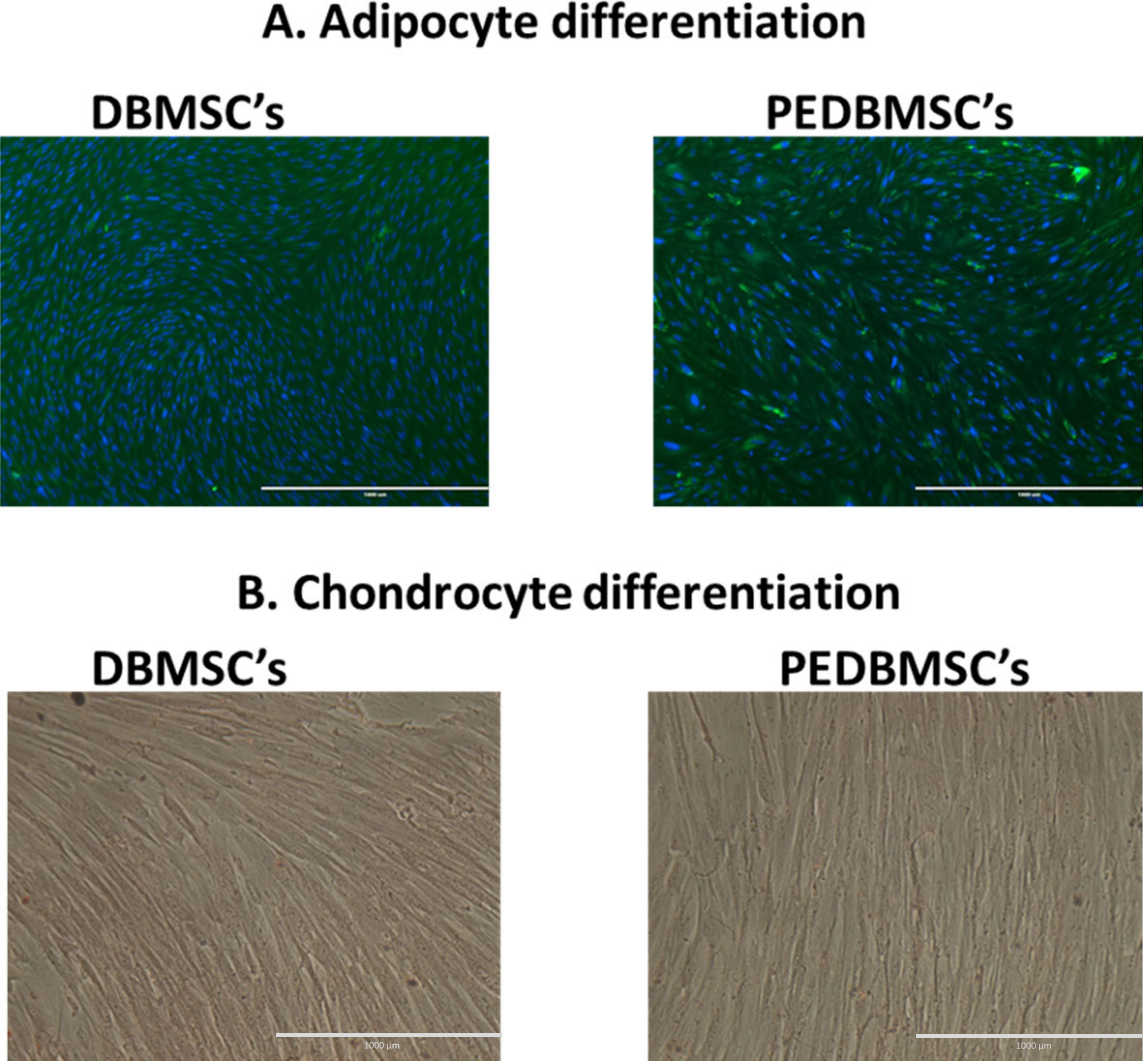
Photomicrographs showing representative examples of PE-DBMSCs at passage 3 (**a**), differentiation of PE-DBMSCs isolated from passage 3 into adipocytes as demonstrated by HCS LipidTOX green neutral lipid staining after 21 days, and (**b**) chondrocytes shown by Alcian Blue staining of a cross-section of a chondrogenic pellet after 21 days

To determine whether PE-DBMSCs differentiate into multiple mesenchymal cell lineages, DBMSCs were cultured in adipogenic and chondrogenic medium. The qualitative confirmation of differentiation was made by LipidTOX Green Neutral Lipid Stain for adipogenic differentiation and Alcian Blue staining for chondrogenic differentiation. As shown in Fig. 1a, b, PE-DBMSCs successfully differentiated into adipocytes and chondrocytes, proving the stemness of these cells.

### Differential expression of immune molecules in PE-DBMSC

To evaluate the differences in the gene expression between the PE-DBMSCs and the normal DBMSCs, a variety of immune molecules were studied by flow cytometry and expression was recorded as median fluorescence intensity. A number of molecules as shown in Table 1 were found to be differentially expressing in the PE-DBMSCs as compared to the normal DBMSCs. They comprised of pro and anti-inflammatory cytokines and chemokines and adhesion molecules. As shown in Table 1, PE-DBMSCs showed decreased levels of some pro inflammatory cytokines and chemokines, and increased levels of anti-inflammatory cytokines and adhesion molecules.

**Table 1:** Differential gene expression in PE-DBMSCs as compared to normal DBMSCs

Decreased levels in PE-DBMSCs		Increased levels in PE-DBMSCs	
Pro-inflammatory cytokines	Pro-inflammatory chemokines	Anti-inflammatory cytokines	Adhesion molecules
IL1A	CCL3	IL10	ITAG 1
IL1B	CCL17	IL19	ITAGA2B
IL6	CCL18	IL20	ITGB2
IL18	CCL24	IL22	ITGAX
1 L21	CCL27	IL10RA	PECAM
IL31	CXCL11	IL20RA	
IL33			

*Abbreviations*: *DBMSCs* decidua basalis mesenchymal stem cells, *PE-DBMSCs* preeclampsia decidua basalis mesenchymal stem/stromal cells, *IL1A* interleukin 1A, *IL1B* interleukin 1B, *IL16* interleukin 16, *IL18* interleukin 18, *IL21* interleukin 21, *IL21* interleukin 21, *IL31* interleukin 31, *IL33* interleukin 33, *CCL3* chemokine (C-C motif) ligand 3, *CCL17* chemokine (C-C motif) ligand 17, *CCL18* chemokine (C-C motif) ligand 18, *CCL24* chemokine (C-C motif) ligand 24, *CCL27* chemokine (C-C motif) ligand 27, *CXCL11* chemokine (C-X-C motif) ligand 11, *IL10* interleukin 10, *IL19* interleukin 19, *IL20* interleukin 20, *IL22* interleukin 22, *IL10 RA* interleukin 10 receptor alpha, *IL20RA* interleukin 20 receptor alpha, *ITAG 1* integrin 1, *ITAGA2B* integrin alpha 2b, *ITGB2* integrin beta 2, *ITGAX* integrin alpha X, *PECAM* platelet endothelial cell adhesion molecule

### PE-DBMSCs show decreased functional potential

**Fig. 2:**
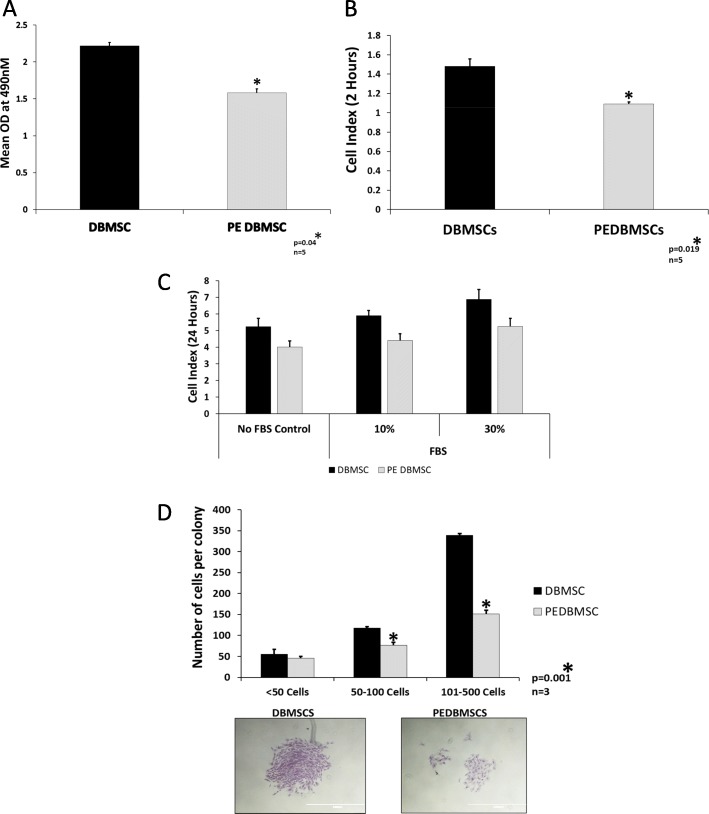
Comparison of functional outcome between PE-DBMSCs and DBMSCs. **a** Proliferation by MTS assay. **b** Adhesion measured after 2 h of the start of the experiment, using the xCELLigence real-time cell analyzer. **c** Migration assay measured using the xCELLigence real-time cell analyzer after 24 h of the start of the experiment. **d** Colony formation assay of DBMSCs and PE-DBMSCs after 14 days in culture. Photomicrographs showing representative examples of a colony-forming units. Experiments were carried out in triplicate using cells at passage #3 prepared from five individual placentae. Bars represent standard errors, *p* ≤ 0.05

For examining the functional potential of isolated PE-DBMSCs, standard functional assays were performed to compare their functional competencies with the normal DBMSCs. These properties were assessed by performing proliferation assays by MTS assay, migration, and adhesion assays by real-time cell analysis assay (xCELLigence), and finally the clone formation assays. As shown in (Fig. 2). PE-DBMSCs were seen to be functionally defective as compared to the normal DBMSCs. They showed decreased proliferation (Fig. 2a), adhesion (Fig. 2b) migration (Fig. 2c) and a significant decrease in clone forming potential (Fig. 2d) as compared to the normal DBMSCs.

### Preconditioning restores the functional properties of PE-DBMSCs

**Fig. 3:**
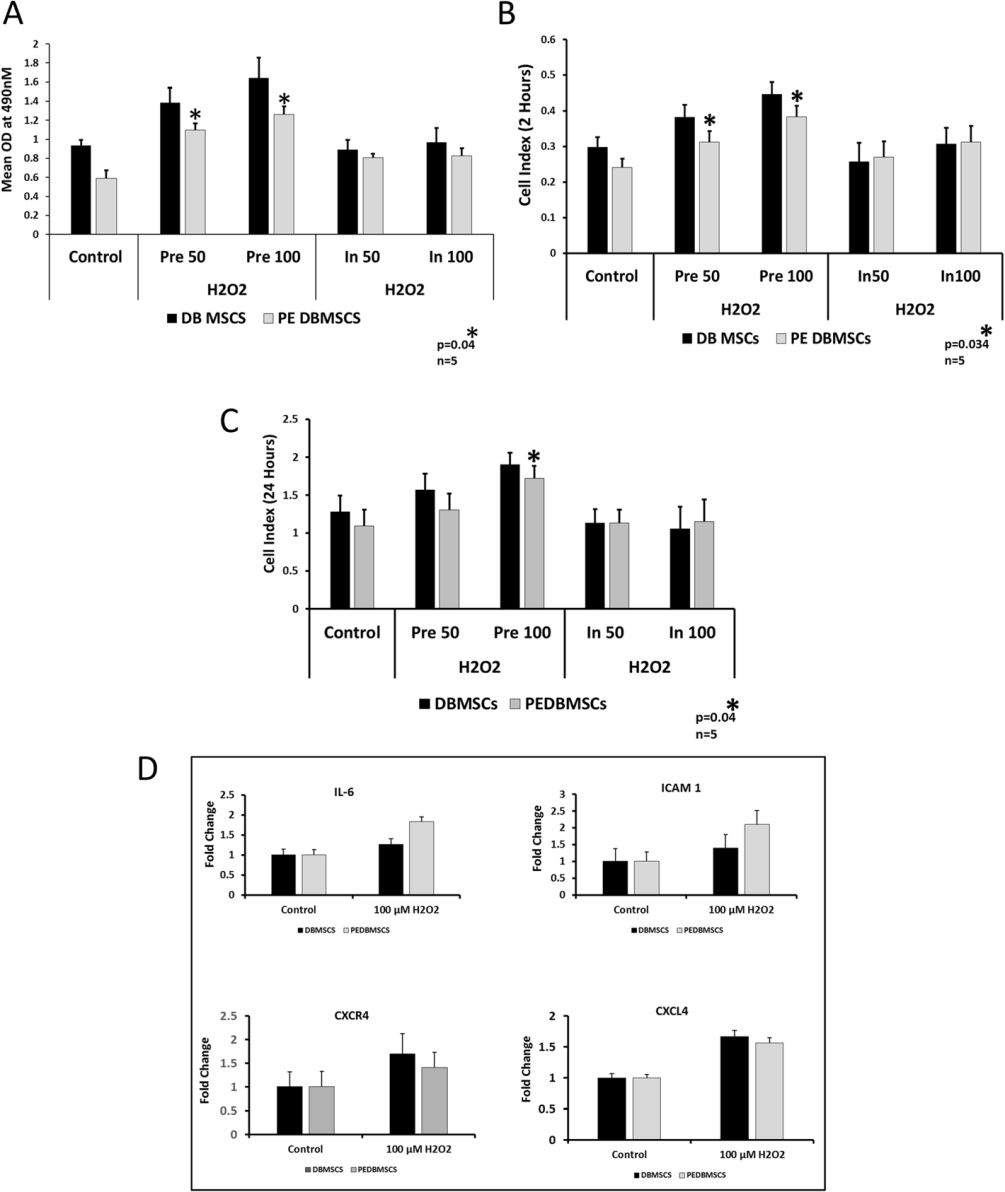
Comparison of functional outcome between PE-DBMSCs and DBMSCs after H_2_O_2_ preconditioning. **a** Proliferation by MTS assay after cells were treated with 50 μM and 100 μM H_2_O_2,_ for 72 h for pre-treatment (Pre) and in-treatment (In) (while performing the assays) testing conditions. **b** Adhesion measured after 2 h of the start of the experiment with pre- and in-treatment conditions using the xCELLigence real-time cell analyzer. **c** Migration assay measured after 24 h of the start of the experiments, while cells were preconditioned with H_2_O_2_ (pre- and in-treatment) using the xCELLigence real-time cell analyzer. **d** RT-PCR analysis of the adhesion molecules, IL-6, CXCR4, ICAM, and CXCL4, after preconditioning with H_2_O_2._ Data is represented as ΔΔ^−2^ (relative fold change) as compared with untreated control. Experiments were performed in triplicate using cells at passage #3 prepared from five individual placentae. Bars represent standard errors, *p ≤ 0.05

We have previously reported that preconditioning enhances the functional properties, such as proliferation, migration, adhesion, and colony formation potential of DBMSCs [14]. To examine if preconditioning with H_2_O_2_ modulates the functionality of PE-DBMSCs and correct their intrinsic defects, we treated PE-DBMSCs with 50 μM and 100 μM of H_2_O_2_ for 72 h (both pre-treatment and in-treatment). We performed multiple functional assays such as proliferation, migration, and adhesion of these pre-treated and in-treated cells. As shown in Fig. 3, preconditioning resulted in a significant increase in functional properties, including proliferation (Fig. 3a), adhesion (Fig. 3b), and migration (Fig. 3c), as compared to the untreated controls. No significant effect was observed in cells with in-treatment sets of experiments. In addition, preconditioning enhanced expression of various adhesion molecules such as ICAM1, CXCR4, IL-6, and CXCL-4 (Fig. 3d).

### HMOX 1 is significantly decreased in a subset of PE-DBMSCs

**Fig. 4:**
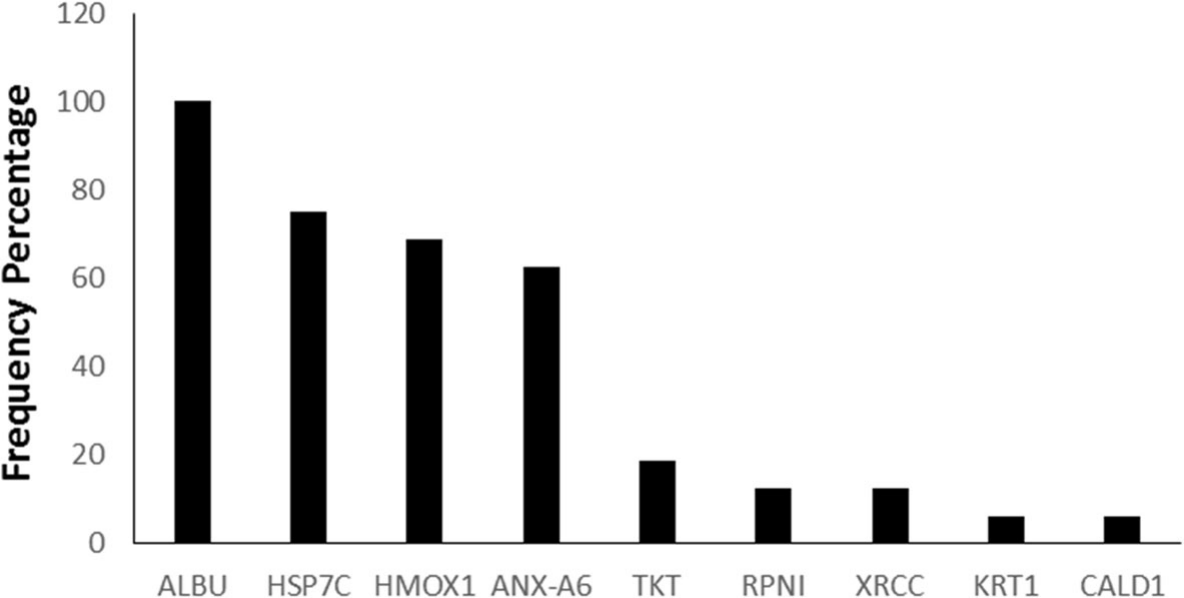
Frequency of the expression of differentially expressed proteins in DBMSCs as compared to PE-DBMSCs. Out of 16 runs in total, the frequency percentage shows the expression levels of different proteins in descending order of expression

**Fig. 5:**
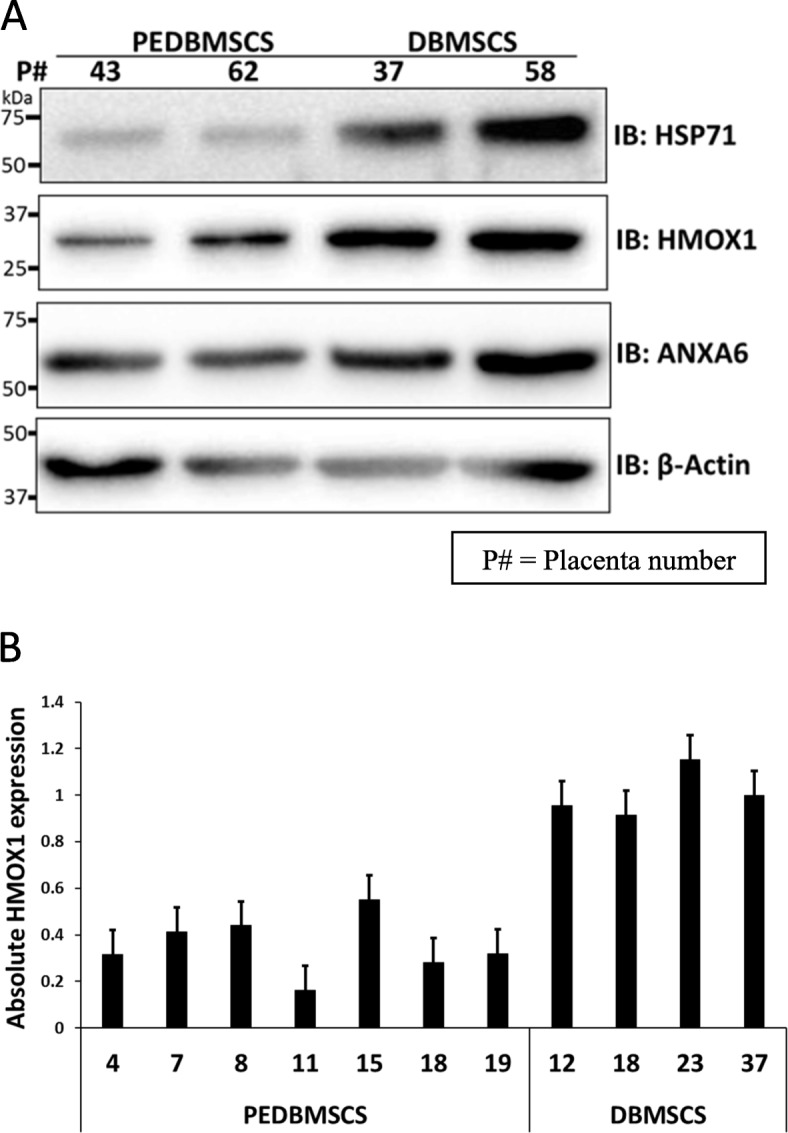
Validation of the mass spectrometry results. **a** Immunoblotting for the major proteins, HSP71, HMOX1, and ANX-A6 in a representative PE-DBMSCs (placenta numbers 43 and 62) as compared to the normal DBMSCs (placenta numbers 37 and 58), respectively. Beta actin is the loading control. **b** RT-PCR analysis of HMOX1 in a subset of PE-DBMSCs as compared to the normal PE-DBMSCs (numbers represent the placenta number tested). Data is represented as ΔcT (absolute expression) as compared with control. Experiments were conducted in triplicate using cells prepared from five placentae. Bars represent standard errors

To identify effectors responsible for differential functional outcomes between the DBMSCs and PE-DBMSCs, we utilized liquid chromatography-mass spectrometry (LC-MS) system to analyze the proteins expressed in DBMSCs and are potentially absent in PE-DBMSCs. The frequency of the presence of the particular protein expressed in DBMSCs and the number of runs performed are shown in Table 2 and Fig. 4. Among the nine confirmed targets, we identified Albumin, HMOX1, HSP71, and ANXA6 most conspicuously absent or downregulated in PE-DBMSCs as conformed by Immunoblotting (Fig. 5a). Among the three proteins, an interesting target HMOX1 expressed in DBMSCs and absent in a large subset of PE-DBMSCs was confirmed by RT-PCR analysis (Fig. 5b) in a large population of PE-DBMSCs isolated from different placentae from PE patients ranging from moderate to severe disease conditions. It was confirmed that HMOX1 either was completely absent or significantly decreased in a wide subset of PE-DBMSCs. However, it was not related to the severity of the disease.

**Table 2:** Differential protein expression in DBMSCs as compared to PE-DBMSCs assessed by mass spectrometry

Name of protein	Total number of runs	Frequency	Frequency (%)
ALBU	16	16	100
HSP7C	16	12	75
HMOX1	16	11	68.75
ANX-A6	16	10	62.5
TKT	16	3	18.75
RPNI	16	2	12.5
XRCC	16	2	12.5
KRT1	16	1	6.25
CALD1	16	1	2.25

*Abbreviations*: *ALBU* albumin, *HSP7C* heat shock protein 7C, *HMOX1* heme oxygenase 1, *ANX-A6* annexin A6, *TKT* transketolase, *RPNI* proteasome regulatory particle subunit, *XRCC* X-ray repair cross-complementing protein, *KRT1* keratin 1, *CALD1* caldesmon

### Blockage of HMOX1 protein decreased functional properties of normal DBMSCs

**Fig. 6:**
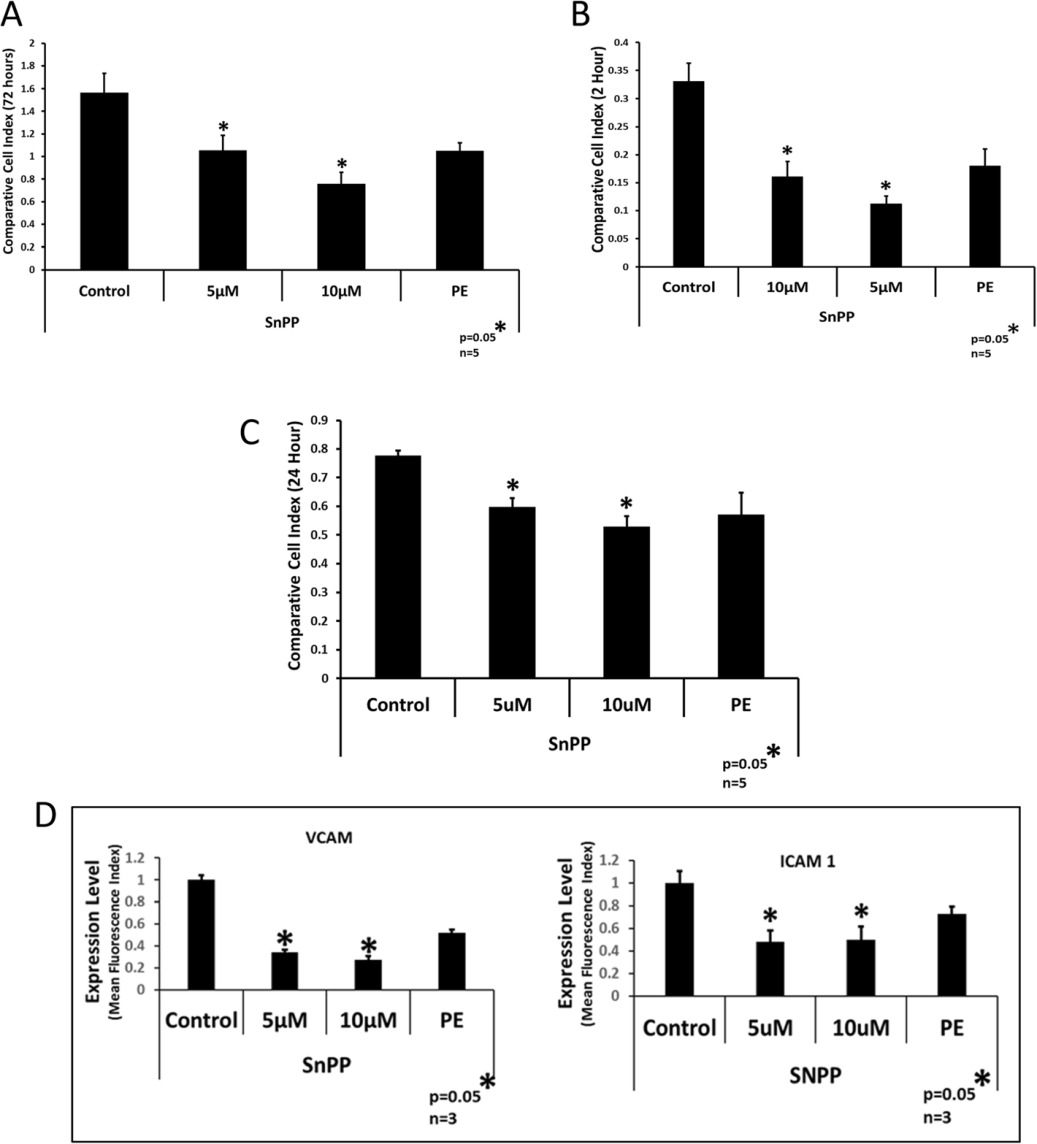
Effect of HMOX1 inhibition by SnPP on functional outcome of DBMSCs. Cells were treated with 5 μM and 10 μM of SnPP for 24 h priors to the start of assays. Cells were tested for **a** proliferation by using the xCELLigence real-time cell analyzer after 72 h of the assay, **b** adhesion assay by xCELLigence real-time cell analyzer measured after 2 h of the start of the experiment, and **c** migration assay measured after 24 h of the start of the experiments, by using the xCELLigence real-time cell analyzer. **d** Flow cytometry analysis of the adhesion molecules, VCAM, and ICAM1, after treating the cells with 5 μM and 10 μM of SnPP for 24 h_._ Experiments were performed in triplicate using cells at passage #3 prepared from five individual placentae. Bars represent standard errors. * represent the significance of the results, ^*^p ≤ 0.05

The functional role of HMOX1 in normal DBMSCs was assessed by blocking its activity using a synthetic heme analog, tin protoporphyrin (SnPP), a selective inhibitor of HMOX1. DBMSCs were treated with two doses of SnPP, 5uM and 10uM for 24 h prior to the start of the functional assays. We tested the effect of HMOX1 inhibition on various functional properties by examining, proliferation, adhesion, and migration of DBMSCs. It was observed that inhibition of HMOX1 activity resulted in the decrease of the functional properties of DBMSCs (Fig. 6a–c). In addition, SnPP treatment reduced the expression of adhesion molecules, such as ICAM1 and VCAM (Fig. 6d), suggesting that HMOX1 may be involved in different cellular processes of DBMSCs.

### Preconditioning enhances expression of HMOX 1 in PE-DBMSCs

**Fig. 7:**
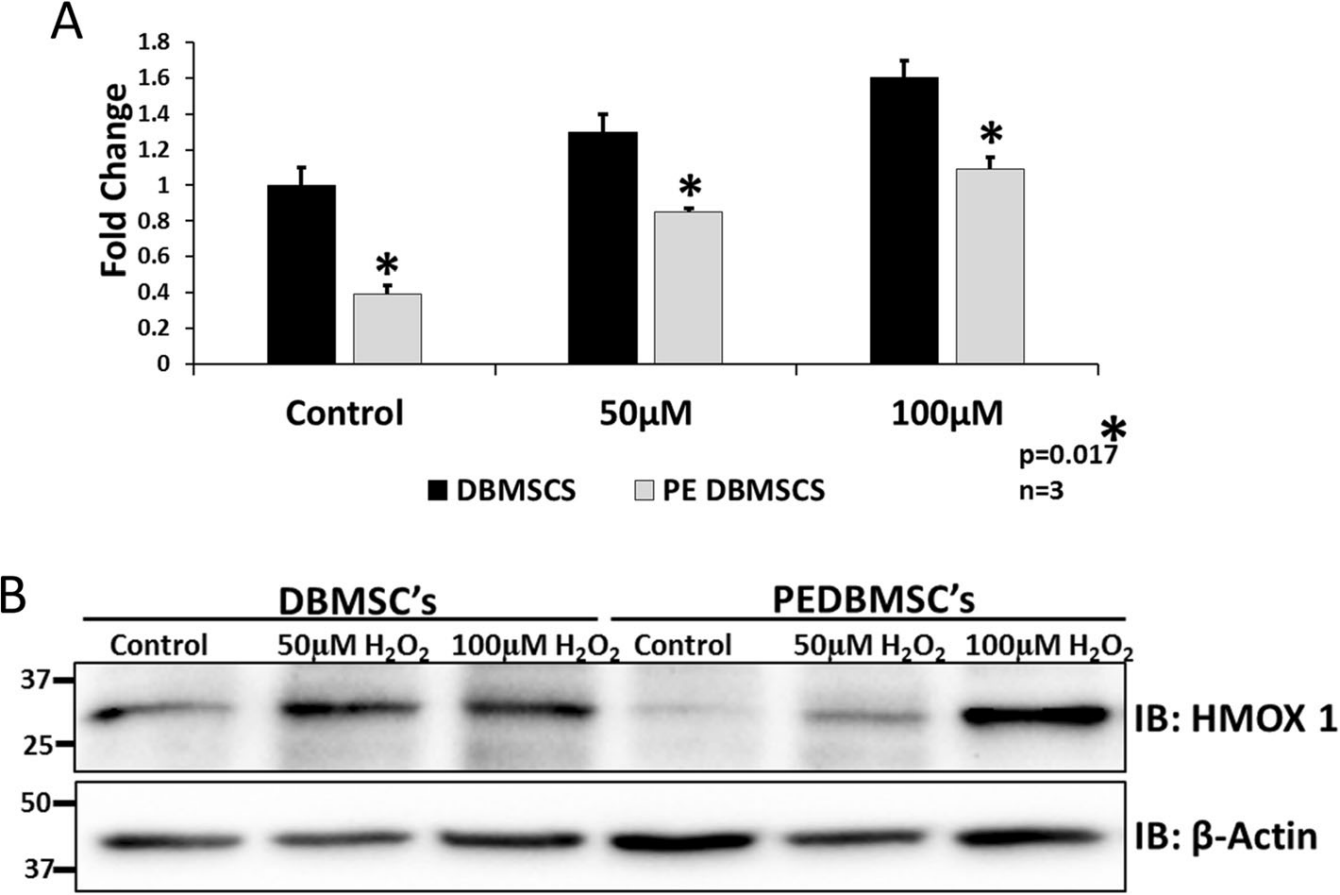
H_2_O_2_ preconditioning enhances the expression of HMOX1 in PE-DBMSCs. **a** RT-PCR analysis of HMOX1 in DBMSCs and PE-DBMSCs preconditioned with 50 μM and 100 μM H_2_O_2_ for 72 h. **b** Immunoblotting for HMOX1 in DBMSCs and PE-DBMSCs preconditioned with 50 μM and 100 μM H_2_O_2_ for 72 h. Beta actin is the loading control. Experiments were performed in triplicate using cells at passage #3 prepared from five individual placentae. Bars represent standard errors. * represent the significance of the results, ^*^p ≤ 0.05

To further study the contribution of HMOX1 in cellular functions in DBMSCs and in PE-DBMSCs, we examined if preconditioning with H_2_O_2_ modulates the expression of HMOX1 in PE-DBMSCs. PE-DBMSCs and normal DBMSCs were preconditioned using 50 μM and 100 μM of H_2_O_2_ for 72 h prior to examining the expression of HMOX1 at mRNA as well as the protein levels. As observed in Fig. 7a, b, preconditioning enhanced the expression of HMOX1 in a dose-dependent manner in both normal and in PE-DBMSCs proving that the increase in functional properties of PE-DBMSCs is mainly dependent upon upregulation of HMOX1 expression. Moreover, H_2_O_2_ preconditioning enhances these properties via the HMOX1 pathway.

## Discussion

We have successfully isolated MSCs from the decidua basalis tissue from the placenta of human patients with PE. These PE-DBMSCs were cultured for up to five passages, depending upon the severity of the disease of the patient, from which this placenta was collected. However, some MSCs isolated from very severe patients did not survive for more than three passages. Morphologically, these cells resembled their normal counterparts and formed a homogenous monolayer of adherent cells that exhibited fibroblast-like characteristics on plastic. Like the normal DBMSCs, these PE-DBMSCs were positive for all the stem cell markers including the HLA-ABC and were negative for all the hematopoietic markers, the endothelial cell marker, co-stimulatory molecules, and HLA-DR. Expression of these molecules remained normal without any change, throughout the culture. However, PE-DBMSCs expressed differentially an array of pro and anti-inflammatory cytokines, chemokines and adhesion molecules (Table 1) as compared to the normal DBMSCs. Expression of these important molecules might give a new dimension in their functional outcome, in terms of therapeutic potential. PE-DBMSC differentiated successfully into chondrocytes and adipocytes (Fig. 1), confirming that they retain the properties of stem cells/stromal cells, which are supposed to differentiate into different cellular lineages [32, 33]. These results confirmed that PE-DBMSCs met the criteria of defining their multipotency, although differing with the normal DBMSCs.

For their successful utilization in transplantation and other therapeutic functions, it is important to study the functional properties of MSCs to evaluate their usefulness and outcome in the disease set up [34]. Since PE-DBMSCs are intimately associated with the endothelial cells that line the vessel wall of the decidua region of the placenta [35], we sought to examine if they play a role in the pathogenesis of the disease itself. While ascertaining the functional properties of PE-DBMSCs, it was noticed that they were functionally defective with reduced proliferation, reduced migration, reduced adhesion and reduced colony formation potential as compared to the normal DBMSCs (Fig. 2). Defective PE-DBMSCs may contribute to the abnormal placentation and vascular remodeling, which has been a hallmark and a necessary component for the genesis of PE [36]. Another characteristic change in the untransformed spiral arteries in PE is acute atherosis. It has been compared to the vascular lesion of atherosclerosis and is claimed to be similar to changes in vessels of tissues undergoing rejection [37]. Atherosis found in disorders other than PE is observed in decidual vessels beyond the site of placental implantation [38]. Endothelial dysfunction is responsible for artherosis-like situation in vascular endothelium, and the PE-DBMSC are located in the close vicinity of the decidua. Diminished functionality of PE-DBMSCs as observed in this study may partly contribute to this situation in the decidua and the endothelium.

We have previously reported that preconditioning with H_2_O_2_ enhances multiple properties of human decidua basalis mesenchymal stem cells/stromal cells [14]. In this study, we conditioned the PE-DBMSCs with two doses of H_2_O_2_ (50 μM and 100 μM) to examine if sustained exposure to H_2_O_2_ for 72 h, increases their functional consequences. Our results showed that functional properties such as proliferation, migration, and adhesion potential of PE-DBMSCs increased significantly after their continued exposure to H_2_O_2_ for up to 72 h. In addition, preconditioning with H_2_O_2_ resulted in the upregulation of adhesion molecules such as IL-6, ICAM1, CXCL4, and CXCR4 justifying the increase in adhesion potential of PE-DBMSCs. Similar results were also reported previously with H_2_O_2_ preconditioning in cardiac progenitor cells [39]; hematopoietic precursor cells [40]; and in MSCs after their exposure to hypoxic and serum deprivation conditions [41].

In addition, to the array of known proteins expressed differently in the PE-DBMSCs and DBMSCs as observed in flow cytometry, we found multiple unknown proteins in a SDS Page Gel Electrophoresis, expressing exclusively in DBMSCs and conspicuously absent or expressing at very low levels in PE-DBMSCs. We sought to identify some of them with the aim to confirm if any of these novel molecules are responsible for decreased functions of PE-DBMSCs. We utilized LC/MS analysis to identify these proteins, and out of them, a few interesting molecules, such as Albumin, HSP71, HMOX1, and ANXA6 as shown in Table 2, were identified. Immunoblotting as well as RT-PCR analysis verified the expression of all these proteins (Fig. 5). Among them, an interesting molecule HMOX1 was seen normally expressing in DBMSCs and completely absent or expressing at extremely low levels in a subset of PE-DBMSCs (Fig. 4).

HMOX is a rate-limiting enzyme, responsible for heme degradation in the ER to generate biliverdin, free iron, and carbon monoxide [16]. It is expressed in two isoforms-HMOX1, a 32 kDa protein is widely distributed in the body, with high concentration in the lung and liver. Whereas HMOX2 is a 36-kDa protein constitutively expressed in the brain, testis, and vascular endothelium [42]. HMOX1 is upregulated with multiple stimuli, such as lipids, hypoxia, hyperoxia, that cause oxidative stress [15]. It regulates important biological functions, such as inflammation, oxidative stress, proliferation, apoptosis, and angiogenesis in various conditions [18].

In pregnancy disorders, such as recurrent miscarriages, intrauterine growth retardation and PE, persistent decreased levels of HMOX1 have been reported [26–28]. MSCs are intimately associated with endothelial cells that line vessel and line the walls in the decidua. PE-DBMSCs with decreased HMOX1 expression as observed in this study justifiably show decreased functional consequences, and they may be a contributing factor for endothelial dysfunction. This property of PE-DBMSCs may play some role in the pathogenesis of this disorder. However, expression of some important molecules such as increased levels of anti-inflammatory cytokines and chemokines, and decreased levels of pro-inflammatory cytokines and chemokines, and increased levels of growth factors as shown in Table 1, may also contribute towards the loss of functionality of endothelium. However, their specific role in the diseased setup such as PE needs investigation.

In order to examine whether decreased levels of HMOX1 was responsible for the loss of function in PE-DBMSCs, we inhibited HMOX1 expression in normal DBMSCs using a synthetic heme-analog, tin protoporphyrin, “SnPP” that selectively inhibits HMOX1 over HMOX2 [43]. Since, HMOX1/carbon monoxide (CO) pathway modulates the expression of soluble Flt-1 (sFlt-1) and soluble Endoglin (sEng) [44], inhibition of HMOX1 activity in DBMSCs by SnPP at 5 μM and 10 μM authenticated the system to be used for other functional assays. While restraining the activity of HMOX1 with SnPP treatment in DBMSCs, the results of functional assays showed a decrease in their proliferation, migration, and adhesion potential as compared to untreated DBMSCs controls. The decrease in ICAM1 and VCAM levels proved the cause of the decrease in adhesion potential of the DBMSCs. Deficiency of HMOX1 is associated with cell damage via apoptosis, necrosis, and inhibition of proliferation and differentiation [45, 46]. HMOX1 deficiency in humans results in severe and persistent endothelial damage marked by elevated thrombomodulin and von Willebrand Factor also [25].

To investigate if the gain of functional consequence in PE-DBMSCs by H_2_O_2_ preconditioning was mediated through HMOX1, we observed that preconditioning by H_2_O_2_ upregulated HMOX1 in both DBMSCs as well as in PE-DBMSCs (Fig. 7) resulting in the enhancement of their functional properties. As explained above, inhibition of HMOX1 by a specific inhibitor resulted in decreased functional outcome in DBMSCs, and phenotypically they resembled the PE-DBMSCs. Upregulation of HMOX1 by preconditioning, followed by the increase in functional consequences, establishes a direct link between preconditioning and functional outcome in both DBMSCs as well as in PE-DBMSCs, which is mediated through and by HMOX1. It has been shown that hydrogen peroxide is an important mediator of intracellular signaling, which potently enhances the expression of HMOX-1 and upregulates synthesis of vascular endothelial growth factor (VEGF) [47]. Loss of HMOX1 in the stem cells/stromal cells may lead to endothelial cell damage and dysfunction, explaining its significant contribution towards the pathophysiology of PE. However, further studies are needed to decipher the cause of HMOX1 inhibition in PE-DBMSCs, and its role in the onset of the disorder.

## Conclusions

This is the first report describing the involvement of the placental mesenchymal stem cells at the onset of PE. We hereby show for the first time that loss of function of stem cells isolated from the patients with PE may contribute towards the disease exacerbation. The mechanism behind this loss of functionality especially in the cellular processes such as adhesion, proliferation, and migration, is demonstrated. HMOX1, a rate-limiting enzyme, which is involved in the important cellular functions, appears to be the strong candidate protein conspicuously absent in PE-derived mesenchymal stem cells/stromal cells. Together, these data suggest that therapies targeted at HMOX1 may prove to be promising for the treatment and management of complicated conditions such as PE and associated diseases. However, their therapeutic significance needs to be validated in preclinical animal studies.

## Data Availability

All the data generated in this study are included in this published article.

## Abbreviations

MSCs: Mesenchymal stem cells/stromal cells
DBMSCs: Mesenchymal stem/stromal cells (MSCs) from the maternal decidua basalis tissue of human term placenta
PE: Preeclampsia
PE-DBMSC: Preeclampsia decidua basalis mesenchymal stem cells
HMOX1: Heme oxygenase 1
SnPP: Tin protoporphyrin
H_2_O_2_: Hydrogen peroxide
IRB: Institutional Research Board
KAIMRC: King Abdullah International Medical Research Centre
MTS: A tetrazolium compound (3-(4, 5-dimethylthiazol-2-yl)-5-(3-carboxymethoxyphenyl)-2-(4-sulfophenyl)-2H-tetrazolium, inner salt
